# Receptiveness and preferences of health-related smartphone applications among Vietnamese youth and young adults

**DOI:** 10.1186/s12889-018-5641-0

**Published:** 2018-06-19

**Authors:** Toan Thanh Thi Do, Mai Dinh Le, Thanh Van Nguyen, Bach Xuan Tran, Huong Thi Le, Hinh Duc Nguyen, Long Hoang Nguyen, Cuong Tat Nguyen, Tho Dinh Tran, Carl A. Latkin, Roger C. M. Ho, Melvyn W. B. Zhang

**Affiliations:** 1 Institute for Preventive Medicine and Public Health, Hanoi Medical University, Hanoi, Vietnam; 2 Department of Hospital Quality Management, Vietnam National Cancer Hospital, Hanoi, Vietnam; 3 Bloomberg School of Public Health, Johns Hopkins University, Baltimore, MD USA; 4 Institute for Global Health Innovations, Duy Tan University, Da Nang, Vietnam; 5 Department of Hepatobiliary Surgery, Viet-Duc Hospital, Hanoi, Vietnam; 6 Department of Psychological Medicine, Yong Loo Lin School of Medicine, National University of Singapore, Singapore, Singapore; 7 Biomedical Global Institute of Healthcare Research & Technology (BIGHEART), National University of Singapore, Singapore, Singapore; 8 School of Medicine and Pharmacy, Vietnam National University, Hanoi, Vietnam

**Keywords:** Smartphone applications, Youths, Young adults, Vietnam

## Abstract

**Background:**

As smartphone becomes increasingly prevalent and affordable, more youths today can own a smartphone device and download applications in various application stores. Smartphone applications have been proven to be useful for youths in various aspects. However, there has been a paucity of data looking into the preferences of Vietnamese youths and adolescents with regards to health-related applications and their receptiveness towards smartphone apps. Therefore, this study aimed to determine the receptiveness and preferences of health-related smartphone applications (mHealth apps) among online Vietnamese youths and adolescents.

**Methods:**

An online cross-sectional study was conducted between the periods of August till October 2015 in Vietnam. Respondent-driven sampling technique (RDS) was utilized to recruit participants. Participants were asked questions about their history of downloading and using health-related smartphone applications and their receptiveness when using these applications. Moreover, socio-demographic characteristics and health status were also self-reported. Multivariate logistic regression was employed to determine associated factors.

**Results:**

Among 1028 participants, 57.4% owned a smartphone and only 14.1% of smartphone users have used a health-related smartphone application, and most of these individuals downloaded the applications for disease prevention (66.3%). 66.4% of the participants who owned these applications reported that health applications were useful and 92.8% reported being satisfied with the functionalities of the applications which they owned. Among smartphone users, people who were employed (OR = 15.46; 95%CI = 4.93–48.47) were more likely to download mHealth apps. Meanwhile, youths with higher EQ-5D index had a lower likelihood of downloading healthcare-related smartphone applications (OR = 0.17; 95%CI = 0.04–0.81).

**Conclusions:**

This study highlighted a low rate of mHealth apps utilization among online Vietnamese youths and adolescents but a high acceptance of individuals who already used these apps. Developing mHealth apps or interventions towards the disease prevention and quality of life improvement could be feasible to proliferate the benefits of such applications in youths and adolescents in Vietnam. Further research should be conducted to optimize the contents and interfaces of mHealth apps that meet the needs of these populations.

## Background

Advances in information technology have led to the advent of the smartphone, which has become an integral part of people’s lives nowadays. A smartphone is a device on which additional features could be added by means of applications, and it is also a device that runs an operating system that allows for various computing options, and enables individuals to be constantly connected to the World Wide Web [1]. According to the data of The Statistics Portal, the number of smartphone users has been predicted to increase from 1.5 billion in 2014 to about 2.5 billion in 2019 [2]. A more recent statistical report has estimated that as of 2017, there are an estimated 6.5 million applications across all the application stores [3]. Approximately 80% of smartphone owners in the United States check their phones every 15 min [4], which implies that individuals are increasingly reliant on these devices to help them in communication, acquisition of information as well as for various other entertainment purposes. Notably, smartphone has a huge number of mobile applications, or “apps”, which are software programs that could enhance the inherent functionality of smartphones. The number of health and medical related applications has been estimated to amount to 100,000 to date, with diverse applications catering to a wide variety of conditions ranging from smoking cessation, to that of weight management in the prevention of common chronic diseases. The vast diversity of apps helps to make smartphones more appealing to the masses, especially for the youths and adolescents. Prior studies have indicated that at least one-fifths of smartphone users have downloaded health related applications on their phone [5] and these individuals have used these applications daily [6].

As smartphone becomes increasingly prevalent and affordable, more youths today are able to own a smartphone. A study in Australia in 2013 found that almost 83% of sampled youths had accessed to smartphone and downloaded an application within the 6 months period [7]. Smartphone could be used to encourage this population to participate in various health and wellness programs [8, 9], such as pain management; weight management; assessment of their dietary and caloric intakes as well as in reproductive health promotion [10–14]. However, the usage of health-related apps is low in this group. Prior research indicated that only 24% of youths owned at least one health-related application and 26% of them have used health related applications only once [15]. The low uptake rates could be attributed to the diversity of the applications available online, as well as the fact that the current health applications conceptualization does not appeal to the needs of the younger generation.

The growth of economy and the affordability of smartphone have resulted in the significantly increasing number of Vietnamese people owning a smartphone in the recent years. A survey in 2016 estimated that 36% of the population have had access to a smartphone device, with a higher penetration rate in the urban areas [16]. Most of owners utilized their smartphones for accessing the Internet and social networking sites [16]. However, there has been a paucity of data looking into the preferences of Vietnamese with regards to health-related applications and their receptiveness towards smartphone applications, particularly among young people. Therefore, this study aimed to determine the receptiveness and preferences of health-related smartphone applications (mHealth apps) among Vietnamese youths and adolescents.

## Methods

### Study design and sampling technique

An online cross-sectional study was conducted from August to October 2015 in Vietnam. Participants were recruited if they met the following inclusion and eligibility criteria: a) Aged between 15 to 24 years old, b) Currently residing in Vietnam and c) Having email or social network account to invite their friends/relatives.

Respondent-driven sampling technique (RDS) was utilized to recruit participants. First, we purposively invited several core groups from universities and high schools in Vietnam (Hanoi Medical University, Vietnam National University, Hung Yen high school, and Phan Boi Chau high school) to participate in the study. These groups were selected to reflect the diversity of sample in consideration of their age, gender and level of educations. All participants were required to provide electronic informed consent prior to the commencement of the online questionnaire study. After completing questionnaire, each participant in the core group was asked to recruit up to five peers or relatives via email or other preferred mass media. The survey was terminated when the network was deemed to be unable to expand further.

Participants were required to provide their answers through the Google Form. Double participants were identified through duplication of email (a total of 7 cases). Individuals who did not meet the eligible criteria were excluded (3 cases). We also excluded people who did not answer more than 60% of the questions in the study. A cumulative total of 1028 participants took part in the online cross-sectional survey.

### Web-survey design

The web-based questionnaire was designed using Google Form. Prior to the commencement of the study, participants were briefly provided information about the purpose of the study, the methodology as well as the information of the principal investigators. The web-based questionnaire consisted of 40 questions, and the participants needed to answer a minimum mandatory number of 23 questions about the primary outcomes of this study and basic socio-economic status. Prior to the actual implementation of the web-based survey, the web-based survey was piloted with twenty youths. The youths assessed the usability of the platform and provided recommendations with regards to the optimization of the web-based survey form. Logical checks for each question were performed. The web-based survey comprises of the following information:

#### Socio-economic status

Including age, gender, education status, occupation, and marital status.

#### Health status

The participants were asked to report whether individuals have had any acute diseases in the last 4 weeks, or any chronic diseases over the last 3 months. In addition, height, weight and body mass index information were also collated to determine whether participants were overweight/obese (≥ 23.0 kg/m^2^). Health-related quality of life was measured by using EuroQol - five dimensions - five levels (EQ-5D-5 L) instrument. The descriptive system includes five domains: Mobility, Self-care, Usual activities, Pain/Discomfort and Anxiety/Depression with five levels of response: no problems, slight problems, moderate problems, severe problems, and extreme problems, giving 3125 health states with respective single indexes. To compute these indexes, the scoring for EQ-5D-5 L was based on the validated scoring done previously in a Thai study [17]. Moreover, we also measured the levels of stress in the last 30 days by using the Short-form Perceived Stress Scale (PSS). This tool assessed four items with a 5-point Likert scale, scoring from 0 (never) to 4 (very often). Total scores were from 0 to 16, which higher scores mean higher levels of stress [18].

#### Smartphone usage and preference

We explored the amount of time individuals spent using their smartphone devices, and the functionalities that they had been using their smartphone for. In addition, we determined whether participants used their smartphone and smartphone applications/mobile services for health-related consultancy (including beauty counselling, nutrition counselling, disease prevention counselling, and disease treatment counselling) on the daily basis. We then examined how individuals were receptive towards the usage of smartphone related health applications and how satisfied they were towards the existing healthcare related applications.

### Statistical analysis

A *p*-value of less than 0.05 was set as the level of statistical significance. We employed a Chi-squared test and Mann-Whitney test to explore the differences of smartphone receptiveness and preference between students and employees. Multivariate logistic regression was used to determine the factors associated with downloading mHealth apps among smartphone users.

## Results

Data in Table 1 revealed that 83.4% of sample was students. A majority of participants were between 18 and 22 years old (64.8%), had undergraduate education (79.4%), were single (72.8%), and living in a homestay (47.7%).

**Table 1:** Baseline demographics information of participants (*n* = 1028)

	Students		Employees		Total	
	n	%	N	%	n	%
Total	857	83.4	171	16.6	1028	100.0
Gender						
Male	335	39.1	89	52.1	424	41.3
Female	522	60.9	82	47.9	604	58.7
Age groups						
< 18	38	4.4	0	0.0	38	3.7
18–22	555	64.8	9	5.3	564	54.9
> 22	264	30.8	162	94.7	426	41.4
Education attainment						
≤ High school	38	4.4	1	0.6	39	3.8
Vocation training	12	1.4	7	4.1	19	1.9
College	52	6.1	7	4.1	59	5.7
Undergraduate	723	84.4	93	54.4	816	79.4
Postgraduate	32	3.7	63	36.8	95	9.2
Marital status						
Single	631	73.6	117	68.4	748	72.8
Having spouse/partner	226	26.4	54	31.6	280	27.2
Current living location						
Homestay	425	49.6	65	38.0	490	47.7
Dormitory	117	13.7	7	4.1	124	12.1
Living with family	232	27.1	84	49.1	316	30.7
Living with relatives	73	8.5	12	7.0	85	8.3

The proportions of participants being overweight/obesity, having acute diseases in the last 4 weeks, and chronic diseases in the last three months were 9.7%, 10.1% and 19.8%, respectively. 8.4% of the students and 16.4% of the employees were noted to be overweight/obesity, and the difference between the two groups of participants was statistically significant (*p* < 0.01). 74.6% reported that they have experienced anxiety or depressive symptoms; and 50.7% of the participants also reported experiencing pain and bodily discomfort. The mean of the EQ-5D index was 0.73. The perceived stress scores of the employees were significantly higher than that of the students group (*p* < 0.05) (Table 2).

**Table 2:** Health Status and behaviors of Participants (n = 1028)

Characteristics	Students		Employee		Total		*p*-value
	n	%	n	%	n	%	
Having acute symptoms in the last 4 weeks							
Yes	88	10.3	16	9.4	104	10.1	0.72
No	769	89.7	155	90.6	924	89.9	
Having chronic diseases in the last 3 months							
Yes	172	20.1	32	18.7	204	19.8	0.69
No	685	79.9	139	81.3	824	80.2	
Overweight and obesity							
Yes	72	8.4	28	16.4	100	9.7	< 0.01
No	785	91.6	143	83.6	928	90.3	
Health related quality of life							
Having problem in mobility	178	19.8	39	21.4	217	20.1	0.62
Having problem in self-care	83	9.2	21	11.5	104	9.6	0.34
Having problem in usual activities	198	22.1	37	20.3	235	21.8	0.61
Pain/Discomfort	458	51.0	89	48.9	547	50.7	0.61
Anxiety/Depression	678	75.5	128	70.3	806	74.6	0.14
	Mean	SD	Mean	SD	Mean	SD	
Height (cm)	1.62	0.08	1.64	0.08	1.62	0.08	< 0.01
Weight (kg)	52.56	8.90	55.45	9.39	53.05	9.04	< 0.01
Body mass index (kg/m2)	19.94	2.20	20.57	2.34	20.04	2.23	< 0.01
EQ-5D index	0.73	0.17	0.73	0.21	0.73	0.18	0.82
Perceived stress score	6.64	2.11	6.27	2.19	6.58	2.13	0.03

Among all the participants, 57.4% owned a smartphone. Only 14.1% of the smartphone users have used a health-related smartphone application, and most of these individuals downloaded the application for disease prevention (66.3%). About 66.4% of the participants who owned these applications reported that health applications were useful and more than 92.8% reported being satisfied with the functionalities of the applications which they owned. Among application-users, nutrition counselling apps had the highest on-apps average time spent per day (18.6 min/day), following by beauty counselling (15.0 min/day) and diseases treatment counselling (15.0 min/day) (Table 3).

**Table 3:** Usage of Smartphone Health & Medical Applications and Attitudes towards Health/Medical applications among participants

	Students		Employees		Total		*p*-value
	n	%	n	%	n	%	
Using smartphone	462	53.9	128	100.0	590	57.4	
Download health-related applications	67	14.5	16	12.5	83	14.1	0.56
Type of applications							
Beauty counselling	34	50.7	13	81.3	47	56.6	0.04
Nutrition counselling	29	43.3	11	68.8	40	48.2	0.06
Disease prevention counselling	42	62.7	13	81.3	55	66.3	0.15
Disease treatment counselling	34	50.7	10	62.5	44	53.0	0.27
Others	9	13.4	1	6.3	10	12.0	0.57
Perceived benefits of health-related applications for health							
Very useful	46	68.7	9	56.3	55	66.4	0.41
Neutral	18	26.9	5	31.3	23	27.7	
Not useful	3	4.5	2	12.5	5	6.0	
Satisfaction with mobile health applications/ services							
Very satisfied	35	52.2	5	31.3	40	48.2	0.26
Neutral	27	40.3	10	62.5	37	44.6	
Dissatisfied	5	7.5	1	6.3	6	7.2	
Time to use mobile phone per day	Mean	SD	Mean	SD	Mean	SD	
For calling (hour)	0.8	1.0	1.2	1.4	0.9	1.1	< 0.01
For texting (hour)	2.4	11.5	1.3	1.6	2.2	10.3	0.23
For others (game, watching movies,…) (hour)	2.6	8.7	2.0	2.2	2.5	7.8	0.50
For beauty counselling (minute) (*n* = 47)	13.2	27.0	21.0	34.2	15.0	28.2	0.49
For nutrition counselling (minute) (*n* = 40)	19.2	68.4	18.0	36.0	18.6	60.0	0.93
For disease prevention counselling (minute) (*n* = 55)	12.6	24.6	20.4	64.2	13.8	36.0	0.43
For disease treatment counselling (minute) (*n* = 44)	16.2	37.8	11.4	16.8	15.0	33.0	0.63

Data in Table 4 showed that 13.0% smartphone users had been overweight/obesity compared to only 5.3% of those who did not own smartphone. Similarly, 14.2% and 32.0% of smartphone owners had had acute symptoms in the last four weeks and chronic diseases in the last three months in comparison with only 4.6% and 3.4% of those not having smartphones, respectively. These differences were statistically significant (*p* < 0.05). Among overweight/obese individuals with smartphone, 24.7% and 32.5% used either a beauty or nutrition counselling application, respectively. Among smartphone users who have had acute symptoms, 42.9% and 39.3% used a disease prevention and treatment counselling application, respectively. Meanwhile, among participants suffering from chronic conditions, 23.8% and 21.1% respectively have used a disease prevention or a disease treatment counselling application (Table 4).

**Table 4:** Usage of Smartphone Health & Medical Applications according to health status

Health status	Having smartphone				*p*-value
	Yes		No		
	N	%	n	%	
Total	590	57.4	438	42.6	
Obesity and overweight					
No	513	87.0	415	94.8	< 0.01
Yes	77	13.0	23	5.3	
• Using beauty counselling app	19	24.7			
• Using nutrition counselling app	25	32.5			
Having acute symptoms in the last four weeks					
No	506	85.8	418	95.4	< 0.01
Yes	84	14.2	20	4.6	
• Using disease prevention counselling app	36	42.9			
• Using disease treatment counselling app	33	39.3			
Having chronic diseases in the last three months					
No	401	68.0	423	96.6	< 0.01
Yes	189	32.0	15	3.4	
• Using disease prevention counselling app	45	23.8			
• Using disease treatment counselling app	40	21.2			

Among smartphone users, people who were employed (OR = 15.46; 95%CI = 4.93–48.47) were more likely to download mHealth apps. Meanwhile, youths with higher EQ-5D index had a lower likelihood of downloading healthcare-related smartphone apps (OR = 0.17; 95%CI = 0.04–0.81). We found no associations between having acute symptoms, chronic disease and body mass index with download mHealth apps (Table 5).

**Table 5:** Factors associated with the use of mhealth apps among smartphone users

Characteristics	Odds Ratio	*p*-value	95% Confident interval	
Age	0.95	0.24	0.87	1.03
Gender (Male vs Female)	0.81	0.48	0.45	1.46
Occupations (Employees vs Students)	15.46	0.00	4.93	48.47
Education attainment (vs < High school)				
• High school	3.67	0.31	0.30	45.24
• > High school	2.77	0.34	0.34	22.65
Marital status (Having spouse/partner vs Single)	1.29	0.43	0.69	2.43
Having acute symptoms (Yes vs No)	1.26	0.62	0.51	3.15
Having chronic diseases (Yes vs No)	1.11	0.76	0.57	2.13
Body mass index (vs Normal)				
• Underweight	0.72	0.46	0.30	1.72
• Overweight/Obesity	1.74	0.31	0.60	5.06
Perceived stress score	0.91	0.14	0.80	1.03
EQ-5D index	0.17	0.03	0.04	0.81

## Discussion

Our current study highlights a low proportion of Vietnamese youths using healthcare related applications, with usage predominantly in disease prevention and beauty counseling applications. On the other hand, we found positive responses among youths using health-related applications, as most of them perceived the usefulness of apps as well satisfied with those apps. These results are important to show the acceptability of health-related smartphone apps and the potential of mobile health intervention among youths in the future.

In this study, more than a half of sample owned a smartphone (57.4%), which was higher compared to this rate in Vietnamese general population [16]. This proportion was lower than that in the developed countries, for example in Hong Kong (86%) [19], South Korea (85%), Japan (65%) [20], or Netherland (90%) [21]; but higher than that of other developing countries such as China (40%) [20] and India (25%) [22]. However, only a minor proportion of smartphone users in our study used medical- or health-related smartphone apps (14.1%). This rate in our study was lower than what found in a study conducted on American adolescents, which found that 21% of smartphone owners used health-related mobile apps [23]. Still, our result was comparable to previous studies that reported a small proportion of smartphone users downloading mHealth apps [16, 24, 25]. The reason for this low rate might be due to the limited availability of healthcare or medical applications in Vietnamese as well as the fact that many of mHealth apps might not be available for free download, lowering the accessibility of the youths to them.

Notably, in our current study, despite the low rates of health-related apps downloaded, among smartphone owners who have downloaded mHealth apps, we have found a slightly higher percentage of individuals who preferred to download and use disease prevention apps compared to other apps. This was consistent with previous surveys, which indicated that fitness, weight management, or tracking health apps were preferred most [26, 27]. Moreover, users seemed to be well-adapted with mHealth apps when more than half of the sample perceived the usefulness of apps and satisfied with these apps. This result is more positive than finding from a previous study conducted in the USA [24], implying the acceptability of youths and adolescents regarding mHealth apps.

In prior studies, education, gender, and age were found as significant predictors of the usage of mHealth apps [12, 19, 23, 26, 28–30], but such relationships were not found in this study. This might due to the relative homogeneousity of these characteristics. Nonetheless, the finding indicated that among smartphone users, individuals who were already employed had a higher likelihood of downloading mHealth apps compared to students. We supposed that employed people might have a higher level of education compared to those who were students; hence, they might have better awareness in how personal health condition impact work performance and general living quality, which promoted them to seek health information, using technological advances such as the Internet or the smartphone. In literature, educational attainment is a significant predictor of health seeking behaviors among young people, such as seeking for further health-related information [28, 31–33].

In this study, despite significant differences between people with and without smartphones regarding health status, associations between having the illness, body mass index, stress and mHealth apps usage were not found in multivariate regression models. This is different from results of previous studies in other countries, which indicated that youths and adolescents having chronic illness were more likely to use mHealth apps to improve their conditions [24, 26]. We assumed that when our respondents suffered from health issues, they tended to visit health facilities rather than downloading and using healthcare apps. Nonetheless, we observed that among smartphone owners, youths having high HRQOL were less likely to download mHealth apps, in other words, people with lower HRQOL had a higher likelihood of using health-related mobile apps. It appears that mHealth apps were used to promote better health status and then improve youths’ quality of life. Therefore, developing mHealth apps that support youths and adolescents in keeping healthy lifestyles and improve their quality of life seems to be reasonable, which can promote the use of mHealth apps in these populations in Vietnam.

There are some limitations of our study which we do need to consider. Our study was a cross-sectional study; therefore, we were not able to track how the attitudes towards health-related smartphone applications would change over time. Secondly, most of the information that we acquired from the sample was self-reported information which may subjected to recall bias as participants might not report their smartphone usage accurately. The perspectives that we have acquired might not be generalizable to the rest of the Vietnamese young population, as the socio-demographics of youths in the general population might be different.

## Conclusion

In conclusion, this study highlighted a low rate of mHealth apps utilization among online Vietnamese youths and adolescents but a high acceptance of individuals who already used these apps. Developing mHealth apps or interventions towards disease prevention and quality of life improvement could be feasible to proliferate the benefits of such apps in youths and adolescents in Vietnam. Further researches should be conducted to optimize the contents and interfaces of mHealth apps that meet the needs of these populations.

## Abbreviation

RDS: Respondent-driven sampling technique
